# Research Models for Studying Vascular Calcification

**DOI:** 10.3390/ijms21062204

**Published:** 2020-03-23

**Authors:** Jaqueline Herrmann, Milen Babic, Markus Tölle, Markus van der Giet, Mirjam Schuchardt

**Affiliations:** 1 Campus Benjamin Franklin, Department of Nephrology, Charité-Universitätsmedizin Berlin, Corporate Member of Freie Universität Berlin, Humboldt-Universtität zu Berlin, and Berlin Institute of Health, Hindenburgdamm 30, 12203 Berlin, Germany; Jaqueline.Herrmann@charite.de (J.H.); Milen.Babic@charite.de (M.B.); Markus.Toelle@charite.de (M.T.); Markus.vanderGiet@charite.de (M.v.d.G.); 2 Department of Chemistry, Biochemistry and Pharmacy, Freie Universität Berlin, Königin-Luise-Straβe 2+4, 14195 Berlin, Germany

**Keywords:** vascular calcification, in vitro, ex vivo, in vivo

## Abstract

Calcification of the vessel wall contributes to high cardiovascular morbidity and mortality. Vascular calcification (VC) is a systemic disease with multifaceted contributing and inhibiting factors in an actively regulated process. The exact underlying mechanisms are not fully elucidated and reliable treatment options are lacking. Due to the complex pathophysiology, various research models exist evaluating different aspects of VC. This review aims to give an overview of the cell and animal models used so far to study the molecular processes of VC. Here, in vitro cell culture models of different origins, ex vivo settings using aortic tissue and various in vivo disease-induced animal models are summarized. They reflect different aspects and depict the (patho)physiologic mechanisms within the VC process.

## 1. Introduction

Cardiovascular disease plays a pivotal role in global morbidity and mortality. One main cause is alterations of the vessel structure, such as atherosclerosis and arteriosclerosis. Arteriosclerosis describes the literal calcification of the media vessel wall of arteries, and atherosclerosis is mainly caused by lipid accumulation and formation of atheromatous plaques in the intima of arteries, with secondary calcification occurring. The calcification in both entities is believed to share underlying mechanisms. Until now, the treatment of vascular calcification (VC) has been limited to management of risk factors with attempts at regulating the impaired calcium–phosphate metabolism. However, VC is an active process which the mechanisms of bone formation, inhibitors of mineralization and local alterations of the vessel wall take part in [1]. One pivotal point of VC is probably the vascular smooth muscle cell (VSMC) with its phenotype changes ending in vessel mineralization [2]. The phenotype shift of VSMC seems to be induced by a variety of conditions such as inflammation [3], reactive oxygen species (ROS) [4,5] and senescence [6]. Aside from differentiated VSMC, other cell types are associated with VC. Mesenchymal osteoprogenitor cells, hematopoietic progenitor cells, endothelial progenitor cells and myeloid cells are circulating cells that bear osteogenic and calcifying potential [7,8]. Not only circulating cells, but also abnormal metabolic conditions such as uremia in the context of chronic kidney disease (CKD) [9], impaired bone metabolism with hyperphosphatemia [10], hypercalcemia and diabetes mellitus type 2 [11,12] lead to medial located calcification, depicting the idea of a systemic disease. The idea of a systemic disease is further supported by decreasing levels of endogenous inhibitors of ectopic calcification like fetuin-a, matrix gla protein (MGP) and inorganic pyrophosphate (PPi) being part of the pathogenesis [13,14]. Under calcifying conditions with high levels of phosphate and calcium in blood, not only cells but also their deposits act as a nidus for the process of mineralization. In order to reduce the intracellular calcium–phosphate burden, VSMC, for example, can form matrix vesicles or apoptotic bodies. Both of these extracellular deposits serve as a nucleation site for hydroxyapatite and therefore promote calcification [15,16,17]. Aside from this, degradation of the extracellular matrix (ECM) by matrix metalloproteinases (MMP) facilitates hydroxyapatite deposition and even osteoblastic transdifferentiation of VSMC [18]. This vast variety of probably influencing factors and different components in the development of VC reflect, at least in part, the variety of research models and vice versa. As long as the underlying mechanisms of VC are not fully understood and treatment options are lacking, evaluation methods and research models will emerge. This review summarizes currently available cell and animal models to study the molecular processes of VC. The assessment and research methods for VC in humans are summarized elsewhere [19].

## 2. In Vitro Models

Our comprehension of processes that underlie VC expands and unravels an intriguing and complex interaction of different cell types and mechanistic signaling. In vitro models are very successful in reducing this complexity and therefore enable scientists to gain insights into the multitude of mechanisms that lead to VC.

### 2.1. Cell Types

Various models allow studying the processes of VC in vitro. Table 1 summarizes the cell types employed to study the mineralization processes of the vasculature with an emphasis on the arterial tree.

VSMC are of particular importance in the calcification of the vessel media: by changing their phenotype from a contractile into an osteoblast-like phenotype, they actively promote VC via different pathways [41]. Therefore VSMC of different origins, including human, rat, mouse and bovine, are by far the most widely studied in vitro model for medial VC [21,22,23,24,25,42]. Next to them, cell lines of murine (MOVAS) and embryonic rat (A7r5 and A10) origin are utilized [28,29,30,31,32].

Myofibroblasts from the adventitia can transdifferentiate bone morphogenic protein-(BMP2)-Msx2 dependently into an osteoblast lineage and contribute to medial calcification [43]. 

Pericytes as progenitor cells have osteogenic potential and can differentiate, among others, into osteoblasts and chondrocytes [44,45]. In pericyte in vitro culture, calcification does not require hyperphospatemia, but takes place in physiological calcium concentration [33].

Endothelial cells (EC) form a monolayer barrier in the intimal layer of the vessel lumen. During development, but also upon vascular injury or several stress factors, EC lose EC-specific markers (e.g., CD31, PECAM-1) and gain expression of mesenchymal progenitor cells—a process referred to as endothelial-to-mesenchymal transition (EndMT) [46,47]. A variety of signaling cascades trigger the EndMT program including, but not limited to, inflammatory conditions and oxidative stress [47]. The transition is characterized by a switch from a fully differentiated phenotype into a pluripotent-like state, where the cell is able to de-differentiate to other mesenchymal-derived lineages. Because of their switch in phenotype, they can accelerate VC progression by secretion of ECM vesicles, expression of adhesion molecules and enhanced proliferation and migration [48]. In addition, they can contribute to ectopic calcification by underging osteogenic differentiation and mineralization [49].

Although this review focuses on calcification of the arteries, cardiovascular calcification can also affect the heart e.g., in aortic valve sclerosis. Here, mineralization is mediated mainly by valve interstitial cells (VIC) that form calcified aggregates when cultured in calcifying medium [40]. VIC can either transform into myofibroblasts or osteoblast-like cells, a process that is triggered by several factors, including calcifying medium and inflammatory stimuli, but also mechanical stress and culture conditions [40,50].

Various circulating cells of mesenchymal or hematopoietic origin can contribute to VC, in particular to intimal calcification, although the underlying mechanisms are not fully understood. In recent years, several contributing cell types were identified and reviewed in detail by Albiero et al. [7] and Cianciolo et al. [8]. The isolation and stimulation procedures of those cells differ. Endothelial progenitor cells derived from rat bone marrow calcify and express markers of osteogenic transdifferentiation after stimulation with oxidized LDL or β-glycerophosphate [51]. In human mesenchymal stem cells, calcification could be induced by dexamethasone or β-glycerophosphate [52]. The contribution of those circulating cells to VC, the driving forces for their shift to calcification and the underlying mechanisms need additional elaboration in functional models.

### 2.2. Stimuli for Calcification In Vitro

Although spontaneous calcification within 6 days of cell culture was shown, for example, for VSMC isolated from spontaneously hypertensive rats [53] and for pericytes [33], most cells studied in in vitro experiments do not calcify spontaneously, but instead require stimuli for calcification. The calcification media used so far vary in the essential components as well as the additional factors (Table 2). Frequently, the medium is supplemented with 5%–20% fetal bovine serum (FBS) in low (1 g/L) or high (4.5 g/L) glucose. Supplementation with phosphate in inorganic (sodium phosphates, 1–5 mM) or organic (β-glycerophosphate e.g., 10 mM) form has a dose- and time-dependent stimulatory effect on calcification and additional supplementation with calcium has an additive effect. Ascorbic acid (AA), a cofactor for a variety of enzymes, is also often added. AA influences mesenchymal differentiation and promotes a phenotype switch of cells [54,55]. By the stimulation of, e.g., type I and IV collagens and MMP-2 activity, AA promotes ECM remodeling [56,57,58]. In addition, the ALP activity increases in the presence of AA and ALP-enriched matrix vesicles contains more apatite-like minerals [58]. Furthermore, sodium pyruvate, insulin or calciferol are frequently used supplements in calcification media. A recent work compared the influence of different factors such as phosphate, calcium and FBS on in vitro calcification yield and reproducibility [59].

### 2.3. Limitations of In Vitro Models

In vitro cell culture experiments are helpful to analyze several research questions e.g., screening substances for inducing/inhibitory effects and/or studying the signaling pathways involved. Nevertheless, one must consider that in cell culture models tissue organization of cells is lost and processes involving cooperative interaction with the ECM cannot be investigated. Research in the field of calcific aortic valve disease gives a striking impression of the effects that non-physiologic environments can have on cell behavior: VIC cultured on a more compliant matrix in calcifying media acquired osteoblast-like properties, while cells cultured on a stiff matrix differentiate into contractile myofibroblasts [40]. Calcification occurred in both cases: on the compliant matrix, calcification was found in aggregates of viable cells that expressed osteoblast-like transcription factors, while on the stiff matrix calcification was found in aggregates containing apoptotic bodies [40]. Alternative models include 3D cell culture models. 3D models mimic the direct physiologic environment and can therefore support physiologic cell behavior, but their implementation is complex. To overcome the problem of myofibroblast differentiation, Hjortnaes et al. developed a 3D model for the research of calcific aortic valve disease that comes closer to the human situation and seems to be a better drug-screening tool than 2D cell culture [61].

## 3. Ex Vivo Models

Beside stimulation of VSMC, ex vivo experiments using vessel tissue are an alternative for studying calcification pathways [62]. Compared to isolated VSMC, the intact vascular cell structure during stimulation procedure is one advantage. Currently, aortic rings from rats and mice are commonly used for studying vessel calcification under various conditions. The stimulation time varies from 3–14 days [6,24,25,28,59,62,63,64,65]. Although the utilization of aortic rings comes closer to the physiologic setting, a multitude of influencing factors is still lost. The luminal side of the aortic ring is not exposed to a flow resembling the blood flow, while the media and adventitia are in direct contact with the calcifying medium or other substances used for stimulation. Endocrine signaling is also missing. To overcome some of these limitations, our group recently developed a modified ex vivo setting of rat artery perfusion in a more physiological way. Here, an increase of mRNA expression in the aortic tissue comparable to in vitro settings and medial-located VC could be induced upon stimulation with high-phosphate medium for 14 days [66]. This model is usable for mouse aortas as well.

## 4. In Vivo Models

In vitro and ex vivo experiments offer a variety of feasible research models for the analysis of VC by providing a detailed insight into one aspect of the broad physiological picture. Nevertheless, an analysis of the whole organism is often required, as in vitro and ex vivo experiments cannot reflect the whole physiological context, but rather individual aspects. In calcification research, rat and mice models have been well established as they offer certain advantages. As calcification is an age-dependent process, rat and mice models enable monitoring of the calcification progression in a reasonable time. The large homology of their genes with humans and the ease of genetic manipulation in addition to their rapid reproduction allow the development of a variety of genetically modified mouse models.

The following chapter will focus on rat and especially mouse models of VC (Table 3). Of course, the application of rat and mice in vivo models also has several disadvantages that will be discussed at the end of this chapter.

### 4.1. Naturally Occuring

Spontaneous and age-dependent mineralization of the vessel wall were found in some animal models. The animals develop sex-specific, mild to moderate medially located VC.

The heterozygous Han:SPRD Cy rat (Cy/+) exhibits a slowly developing and progressive polycystic kidney disease [67] and develops a mild medial-located VC when fed a high phosphate (0.7%) diet [68]. The severity of the kidney damage is sex-specific and progresses predominantly in male rats [67]. In the calcification study, male animals were used [68]. Another rat model suffering from renal failure is the Lewis polycystic kidney disease rat. These animals develop increased arterial stiffness and aortic calcium content compared to Lewis rats with normal kidney function [69].

An age-dependent increase in the severity of soft tissue calcification was also observed in DBA/2 mice [70]. Specifically, cardiac tissue calcification was observed in 6-week-old mice followed by mineralization of the soft tissues in 39–52-week-old mice [70]. The severity of calcification was higher in female than in male animals [70]. Limitations of these models may include the long experimental duration as well as the less progressive medial calcification. However, the mild severity might be a benefit for intervention studies.

### 4.2. Induction of a Disease State by Chirurgic Intervention and Substance Application

Beside the models described above, rodents are not prone to VC, so several intervention procedures are necessary for induction of a certain degree of disease. In line with the calcification progression under uremic conditions in humans, rats and mice develop comparable vessel calcification upon uremic conditions. For induction of chronic renal failure, several protocols exist, mainly by surgical reduction of kidney mass and ureter obstruction or dietary intake of nephrotoxic adenine diet. The most common surgical method is the 5/6 to 7/8 reduction of functional kidney mass in rats and mice [71,72]. Various techniques and procedures have been used in the past: electrocautery or dissection of renal arteries combined with full nephrectomy on the contralateral side in a one-step or two-step surgical procedure [73]. The major limitations of these models are the high effort required for the surgical preparation, the surgery-dependent variation in the CKD (mild to severe) and post-operative complications. Furthermore, comparable vessel calcification occurs after an extended observation period [73].

A dietary component that causes kidney failure is a high dietary intake of adenine [74]. The precipitation of adenine causes nephrotoxicity along the renal tubules and urinary tract [74], which induces severe kidney damage representative of uremic features of the human condition [40,74,75]. The disease progression is associated with moderate to severe aortic calcification localized primarily in the media of the vessel wall [40,75,76,77,78,79,80,81,82,83]. In contrast to the high effort required for the surgically-induced CKD rodent models, the experimental process of generating adenine-induced CKD is relatively easy. However, a limitation of the older treatment protocols is weight loss and the high biological variability in calcification progression. Furthermore, the original model with a 0.75% adenine diet has many confounding factors in researching VC e.g., high blood pressure, lipid disorder, rapid malnutrition and high fatality at 4–6 weeks [84]. Therefore, the administration protocols in various VC studies with rats have been optimized for adenine content, dietary supplements (vitamins, grain/casein), time course of treatment (2–12 weeks) and intervals of standard chow to control food intake and weight loss. Shobeiri et al. reduced the adenine content to 0.25% and characterized the calcification of different vascular beds after 5 to 11 weeks of treatment [76]. In this dosing protocol, extreme weight loss was not an issue (9%–12% reduction of the initial weight) [76]. Furthermore, a progression of increased calcification of various vessel types was found [76]. The adenine-dosing schemes vary between different treatment protocols. Some provided the same adenine content throughout the whole course of the experiment [40,76,85], whereas others changed the adenine content e.g., by dose reduction from 0.75% to 0.5% adenine after one week [86] or an interval period with standard chow [77,81,86]. Between those models, blood parameters such as blood urea nitrogen (BUN), calcium and PTH seem to be comparable. However, weight loss of the animals varies between the studies. Interestingly, the severity of the vascular medial calcification is exceptionally variable. One study found moderate to severe mineralization in different vascular beds of Wistar rats upon feeding a chow with a constant 0.25% adenine content for 5 to 11 weeks [76]. In contrast, no VC was found in Wistar rats using a dose-reduction adenine regime (0.75% adenine diet for 2 weeks, followed by 2 weeks of 0.5% adenine chow and subsequent standard chow for 5 weeks) [86]. Inferentially, the extent of VC depends on the duration and dose of adenine administration. Until recently, the adenine-feeding model has only been used in mineralization studies with rats, because a high adenine content in mice causes acute kidney failure with a mortality rate of nearly 100% within 6 days [87]. Recently, Santana et al. characterized a mouse model for CKD using a 0.2% adenine diet for up to 6 weeks [85].

To answer the question if potential differences in CKD conditions using surgical or adenine-diet protocols exists, one laboratory compared the 5/6 nephrectomy rat model with the adenine-diet-induced CKD, while animal and housing conditions were consistent. The authors observed no differences in the blood parameters including serum phosphate, calcium, PTH and fibroblast growth factor 23 (FGF23). However, the adenine-fed rats had a higher rate of bone turnover [86].

In addition to kidney failure, diabetes mellitus is also associated with VC. To study diabetic artery calcification, a model with rats combining streptozotocin-induced diabetes (SD) with high-fat diet and vitamin D3 treatment was established [88].

Additionally to disease induction in rodents, special dietary supplements are necessary to enhance the effects. For example, the SD rat strain was resistant to VC when fed a diet without increased phosphorus content [89]. Other authors have used various phosphorus concentrations (0.6% to 1.8%) combined with dietary calcium contents ranging from 0.6% to 4% as well as supplementation with various amounts of vitamin D [86,89,90,91,92,93,94,95,96,97,98,99,100,101,102,103]. The extent of arterial mineralization depends on duration of treatment and the diet used. Depending on the genetic background of the animal, the dietary regime and degree of kidney damage, the severity and location of VC varies. Most of the studies involved increased dietary calcium and phosphorus content. Moreover, supplementation with dietary vitamin D [104,105,106,107] and cholesterol [105] has been found to enhance arterial VC in various rodent studies. However, vitamin D administration influences physical impairment and promotes weight loss [105,107]. The cholesterol-enriched diet was primarily used in models studying intimal calcification. Furthermore, nicotine [88,108,109,110] has been administrated to animals to induce or promote mineralization of the vessel wall.

In addition to the diet-induced effects on VC, a recent study in uremic rats investigated the influence of electromagnetic fields on promoting VC [111]. However, no effects of electromagnetic fields were found in rats without kidney damage [111].

### 4.3. Genetically Modified Mouse Models

In genetically modified mouse models, atherosclerotic plaque formation and calcification can be reinforced by manipulation of cholesterol metabolism. Calcification of the media can be amplified by interruption of vascular protective mechanisms, which physiologically inhibit calcification or increase serum phosphate concentration by uncoupling of physiological phosphate metabolism.

#### 4.3.1. Phosphate Metabolism

**Figure 1:**
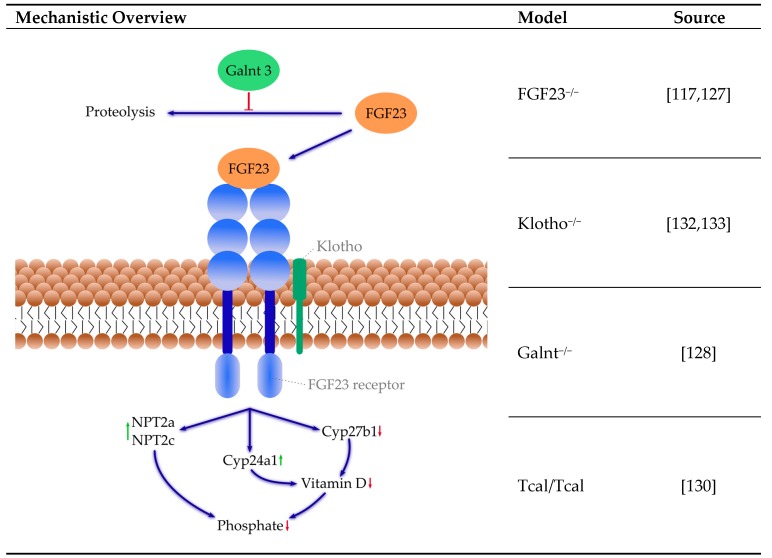
Selected mice models of altered phosphate metabolism and their mechanisms. Green arrows: activation, red arrows: down regulation, blue arrows: pathway. References: [117,127,128,130,132,133]. Cyp24a1: 24-hydroxyvitamin D-1α-hydroxylase; Cyp27b1: 25-hydroxyvitamin D-1α-hydroxylase; FGF23: fibroblast growth factor 23; Galnt3: N-acetylgalactosaminyltransferase 3; NPT2a: renal sodium-dependent phosphate co-transporter type IIa; NPT2c: renal sodium-dependent phosphate co-transporter type IIc.

Elevated phosphate can be a consequence of increased phosphate absorption, decreased phosphate excretion or a shift from intracellular to extracellular phosphate. Hormonal regulation of phosphate involves the intestine, kidneys and bones and several signaling pathways, including, but not limited to, vitamin D, FGF23 and α-Klotho. Disturbance in vitamin D and calcium–phosphate metabolism was shown to play a role in the progression of ectopic mineralization [112,113]. Reduction in active vitamin D, by either vitamin D deficient diet, disruption of the vitamin D receptor, or its gene reduced ectopic calcification. However, these interventions likewise reduce phosphate blood concentration, whereas phosphate reduction, even when increasing active vitamin D and calcium concentration, is effective in reducing VC [112,114,115,116,117,118,119,120,121]. Therefore, the following models are summarized as altered phosphate metabolism (Figure 1).

**FGF23**: Hyperphosphatemia often succeeds CKD, hypoparathyreoidism and vitamin D intoxication, but can also result from rare genetic disorders like hyperphosphatemic tumoral familial calcinosis (hFTC). In hFTC, the FGF23 receptor cannot be activated due to a mutation of either the FGF23, the α-Klotho or the GalNAc transferase 3 (GALNT3) gene [122]. FGF23 is an essential regulator of phosphate homeostasis and vitamin D metabolism promoting phosphaturia. FGF23 decreases the surface expression of sodium-dependent phosphate co-transporters type IIa (NPT2a) and IIc (NPT2c) in the proximal renal tubules. Furthermore, it reduces vitamin D availability by downregulation of the expression of the Cyp27b1 gene, encoding 1α-hydroxylase, an enzyme required for active vitamin D synthesis. Furthermore, the expression of the Cyp24a1 gene is upregulated, which encodes vitamin D degrading 24α-hydrolase [120,123,124,125,126]. Thus, FGF23 suppresses synthesis and promotes degradation of vitamin D. Mice lacking FGF23 exhibit increased plasma levels of phosphate and calcium [115,127] as well as VC [115,117]. Interestingly, whereas a phosphate-deficient diet prevented vessel calcification in the FGF23^−/−^ mice, a vitamin D deficient diet did not indicate a significant role for hyperphosphatemia in that calcification model [115]. In addition, the mice lacking FGF23 exhibit a premature aging process similar to Klotho^−/−^ [117]. The aging process seems to be partly exerted through effects on vitamin D metabolism because a genetic ablation of the 1α-hydroxylase reduced the aging phenotype in the FGF23^−/−^ mice [117]. O-glycosylation of FGF23 through Galnt3 reduces the susceptibility of FGF23 to proteolysis and therefore permits the secretion of intact FGF23 [128].

**Galnt3**: Patients with mutations in Galnt3 suffer from hyperphosphatemia and extensive calcium depositions [129]. Results from mice models are contradicting: Galnt3^−/−^ mice have hyperphosphatemia and increased FGF23 expression, although secretion of intact FGF23 is impaired, but show no sign of abnormal calcification [128]. In contrast, Tcal/Tcal mice, which feature a missense mutation in the Galnt3 gene, feature hyperphosphatemia and extensive ectopic calcification [130]. In Galnt3^−/−^ mice, breeding with FGF23 transgenic mice can increase the amount of intact FGF23 and reduce hyperphosphatemia [131].

**Klotho**: Klotho is a necessary co-factor required for FGF23 binding to its receptor. Because most tissues and cells express FGF receptors, the target organs of endocrine FGF are determined by tissue-specific expression of Klotho [134]. Klotho deficiency results in high FGF23 levels [112,135]. Similarly to the FGF23^−/−^ mice, mice deficient in the Klotho gene show ectopic soft-tissue and vessel mineralization and premature aging [132]. In Klotho/1α-hydroxylase double knockout mice, VC and soft tissue calcification were eliminated [118]. In the absence of membrane-bound Klotho, delivery of circulating soluble Klotho reduced serum phosphate levels and aorta mineral content in alpha Klotho null mice [133].

#### 4.3.2. Absence or Dysfuntion of Calcification Inhibitor Proteins

**Figure 2:**
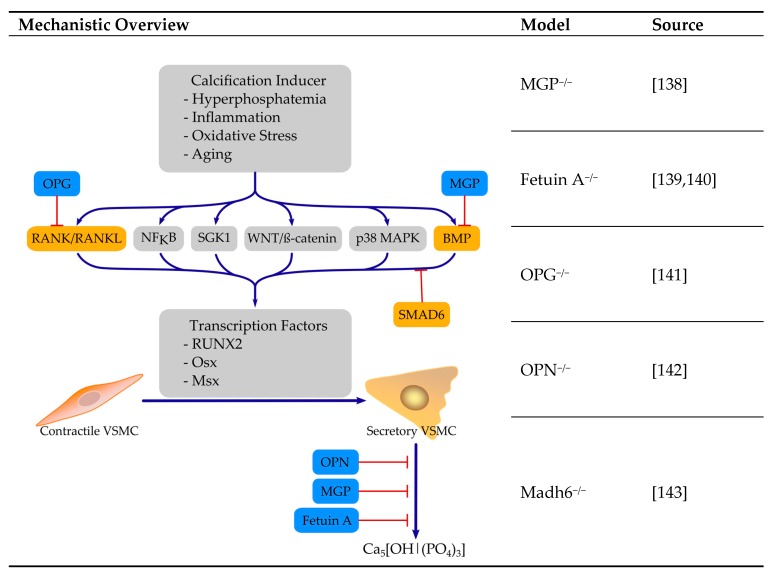
Selected mice models with absent or dysfunctional calcification inhibiting peptides and their mechanisms. Red T-arrows: inhibition, blue arrows: pathway. References: [138,139,140,141,142,143]. MGP: matrix-gla protein; OPG: osteoprotegerin; OPN: osteopontin; Madh6: gene coding for smad family member 6.

Mineralization is a tightly controlled process and several mechanisms serve as inhibitors of ectopic mineralization (Figure 2). Body fluids tend for mineralization as they are supersaturated in phosphate and calcium. Several endogenous circulating calcification inhibitors help to prevent pathophysiological mineralization. In addition, promotors and inhibitors of calcification could also serve as biomarkers for onset and progression of VC [136]. Mouse models with disrupted protective mechanisms partially feature extensive ectopic calcification. Circulating inhibitors prevent calcification by different mechanisms and include MGP, Fetuin-A, osteoprotegerin (OPG), osteopontin (OPN) and PPi, which as a non-peptide inhibitor of VC is considered later. The consensus statement of Bäck et al. [137] offers an excellent overview of the endogenous calcification inhibitors and their therapeutic potential.

**MGP**: In mice, deletion of the MGP gene is lethal, due to extensive VC [138]. Death occurs within two months of age because of thoracic or abdominal aortic rupture [138]. In these mice, VSMC undergo chondrocyte differentiation and form cartilage in blood vessels [144]. The arterial phenotype of MGP-deficient mice can be restored in a transgenic animal, where MGP is reintroduced in VSMC [145]. The molecular mechanisms by which MGP prevents ectopic calcification include prevention of mineralization by binding of calcium ions as well as inhibition of the pro-osteogenic effects of BMP2 (reviewed in detail by Proudfoot and Shanahan [146]). MGP requires vitamin K dependent γ-carboxylation to exert its calcification inhibitory functions. Therefore, models of vitamin K inhibition e.g., by application of warfarin, exhibit similar calcification of the arteries [147].

**Fetuin A**: Fetuin A, also known as α_2_-Heremans-Schmidt glycoprotein (ahsg), inhibits ectopic mineralization through the formation of fetuin-mineral complexes, which are also termed calciprotein particles [148,149]. Mice on a C57BL/6-129sv background deficient for fetuin show no general ectopic calcification, although serum inhibition of apatite formation is diminished and some homozygous animals develop soft tissue calcification after breeding [139]. Feeding these animals with a chow rich in minerals and vitamin D resulted in an increase in calcification and backcrossing these mice to the more calcification-prone DBA/2 background resulted in severe systematic calcification [140].

**OPG**: OPG is a soluble decoy receptor for the receptor-activated nuclear factor κB ligand (RANKL), also known as tumor necrosis factor receptor superfamily member 11B (TNFRSF11B). As one of its mechanisms, it prevents interaction of RANKL and RANK, thus inhibiting downstream signaling such as osteoclastic cell differentiation, survival and function [150]. Mice deficient for OPG exhibit calcification of the aortic media and renal arteries [141]. When mice deficient for OPG were treated with injected recombinant OPG, the incidence of aortic calcification was not reduced, whereas transgene animals showed no calcification of major arteries [151].

**OPN**: OPN, also known as secreted phosphoprotein 1 (SPP1), is a multifunctional protein that also serves as a mineralization inhibitor and is found in abundance at sites of mineral calcification, but the precise mechanisms of action remain unclear to date [152]. Deficiency in OPN alone is not sufficient to induce spontaneous VC, but the deficiency in OPN combined with other inducers of VC e.g., deficiency in MGP, reinforces mineralization [142].

**SMAD6**: Smad6 is an inhibitor of tumor growth factor β (TGFβ) signaling and a negative regulator in BMP signaling [153,154]. Targeted deletion of Madh6, the gene that encodes Smad6, results in a mice model that experiences cardiovascular mortality, including aortic ossification, which is restricted to areas with VSMC and increases lethality [143].

Taken together, these mice models exhibit particular conditions for calcification. They have been very critical in the identification of pathophysiological mechanisms resulting in ectopic calcification and have helped to move the perspective of VC as a tightly controlled active process.

#### 4.3.3. Pyrophosphate System

**Figure 3:**
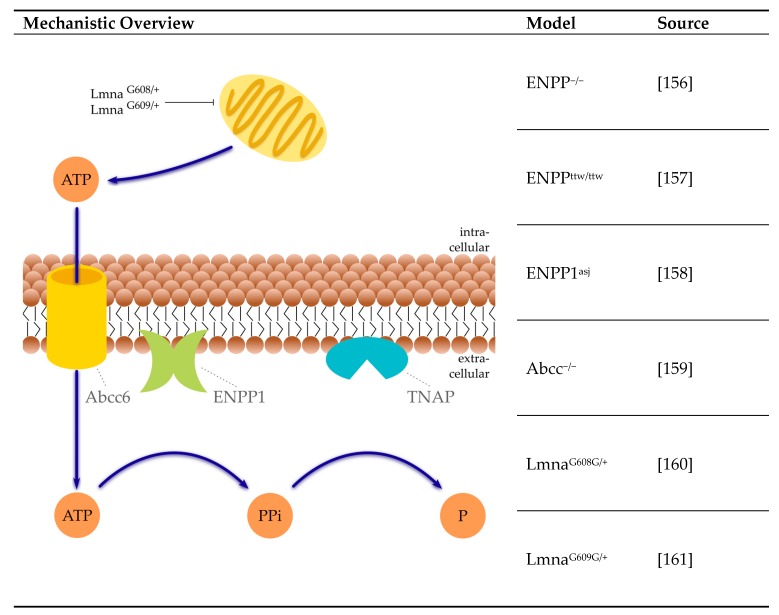
Selected mice models of reduced serum pyrophosphate concentration and their mechanisms. Blue arrows: pathway. References: [156,157,158,159,160,161]. Abcc: adenosinte tetraphosphate-binding cassette subfamily C; ATP: adenosine tetraphosphate; ENPP1: ectonucleotide pyrophosphatase phosphodiesterase; Lmna: lamin A/C gene; P: phosphate; PPi: inorganic pyrophosphate; TNAP: tissue-nonspecific alkaline phosphatase.

PPi is a crucial circulating inhibitor of VC preventing calcium apatite precipitation [155]. Serum PPi can have several sources (Figure 3).

The hydrolysis of ATP by ectonucleotide pyrophosphatase phosphodiesterase (ENPP1) forms the majority of PPi [14]. A significant part of ATP is provided by the release from hepatocytes via ATP binding cassette subfamily C member 6 (Abbc6) [162]. Next to that, PPi can be directly transported from the intracellular to the extracellular environment via ANK [163]. The major sources for ATP are the mitochondria. Therefore, mitochondrial dysfunction can reduce the available amount of ATP, as, for example, in the rare genetic human disorder Hutchinson–Gilford progeria syndrome. A number of other orphan diseases in humans feature reduced circulating PPi levels, resulting in ectopic calcification, including generalized arterial calcification of infancy and pseudoxanthoma elasticum.

**ENPP1**: The enzyme ENPP1 generates extracellular PPi from ATP. Mice lacking ENPP1 are prone to the development of VC. Different mice models for ENPP1 deficiency currently exist, including the genetically engineered ENPP1^−/−^ mice, the tip-toe walking mice (ENPP^ttw/ttw^) and the mutant ENPP1^asj^ mice [156,157,158]. All three mice models suffer from VC of the aorta. From these models, ENPP^−/−^ mice are best studied. In ENPP1^−/−^ mice, aortic calcification was developed within 2 months of age. Calcification is accelerated by phosphate diet [14]. However, under the same chow, aortas transplanted from ENPP1^−/−^ mice into WT mice did not calcify, which indicates that the systemic availability of sufficient amounts of PPi is sufficient to prevent calcification [14]. On a high phosphate diet, ENPP^ttw/ttw^ mice exhibit ectopic aortic calcification associated with increased aortic Runx2 expression [157]. ENPP^asj^ mice also demonstrate an early onset of extensive arterial calcification upon being fed a high phosphate diet [158].

**Abcc6**: In Abcc6^−/−^ mice, arterial calcium accumulates at the age of 16 months and was around 2-fold higher than in wild type mice [159]. Administration of PPi and etidronate inhibited calcification, but was unable to reverse already existing calcification [164]. Similar results were found in a study where early intravenous administration of a wild type human Abcc6 expressing adenovirus into Abcc6^−/−^ mouse reduced mineral deposition, but late administration failed to reduce mineralization [165].

**Lamin A/C (LMNA)**: Mutations in the LMNA gene result in the synthesis of progerin, a splicing isoform of the precursor protein prelamin A [166]. Alternatively, a mutation in ZMPSTE24 (farnesylated protein-converting enzyme 1) can result in an abnormal accumulation of prelamin A [166]. In G608G transgenic mice (Lmna^G608G/+^), pathological changes in the media layer of large vessels e.g., VSMC loss and calcification [160] have been reported. In heterozygous Lmna^G609G/+^ mice, increased medial calcification of the aortic arch and thoracic aorta, as well as reduced PPi levels, were found, resulting from impaired mitochondrial ATP synthesis [161].

#### 4.3.4. Lipoprotein System

**Figure 4:**
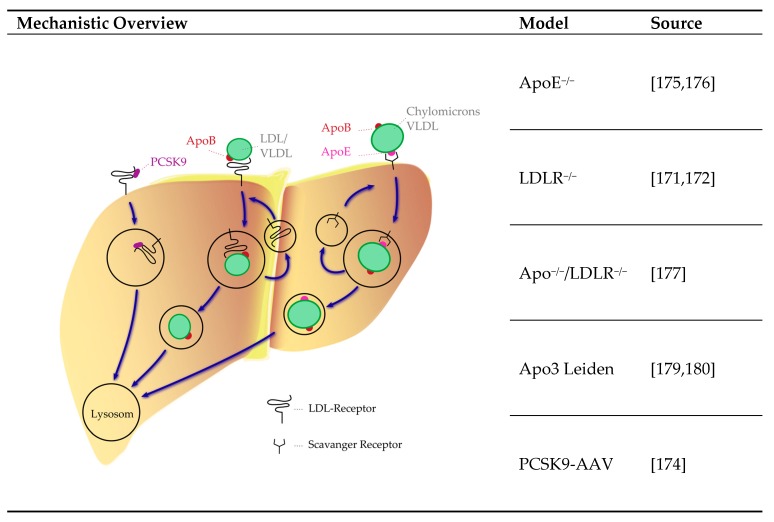
Mice models of atherosclerotic hyperlipidemia and their mechanisms. Blue arrows: pathway. References: [171,172,174,175,176,177,179,180]. AAV: adeno-associated virus vector; Apo: Apolipoprotein; LDL: low-density lipoprotein; PCSK9: pro-protein convertase subtilisin/kexin type 9; VLDL: very low-density lipoprotein.

Hyperlipidemia in one of the main triggering factors in the pathophysiology of human atherosclerosis. In mice, the lipoprotein profile significantly differs from humans [167,168]. Mice are lacking the cholesteryl ester transfer protein, an enzyme that transfers cholesterol from high-density lipoprotein (HDL) to apolipoprotein B-containing lipoproteins as low-density lipoprotein (LDL) and very low-density particles (VLDL) [169]. In addition, mice also have bile acid compositions that differ from humans [168,170], affecting the enterohepatic cycle of cholesterol [170]. Several models for the disruption of lipid metabolism exist e.g., including LDL receptor (LDLR)^−/−^, apolipoprotein E (ApoE)^−/−^, LDLR and ApoE double knockout, ApoE3 Leiden transgenic mice and proprotein convertase subtilisin/kexin type 9 (PCSK9) gain of function models (Figure 4). Additional existing models are reviewed in detail elsewhere [167].

**LDLR and PCSK9**: LDLR dysfunction results in the accumulation of LDL in patients with familial hypercholesterolemia. Mice homozygous for LDLR deficiency have a delayed clearance of VLDL and LDL from plasma and calcifications of the aorta [171,172]. Overexpression of PCSK9 results in an increased degradation of LDLR [173]. When mice are injected a gain of function PCSK9 adeno-associated virus vector (AAV), they develop increased cholesterol levels, atherosclerotic lesions and aortic calcification [174].

**ApoE**: Clearance of remnants of chylomicrons and VLDL require ApoE as a receptor ligand. Knockout of ApoE results in atherogenic accumulation of cholesterol-rich remnants [175] and development of calcification of advanced lesions [176]. ApoE and LDLR double knockout mice developed calcifications along the aortic arch [177]. Inducing uremia by partial kidney ablation in ApoE^−/−^ mice resulted in an acceleration of arterial calcification [178]. A mutation in the ApoE gene can result in a defective protein, which has reduced capacity for remnant clearance [179]. In transgenic Apo3 Leiden mice, a high fat/cholesterol diet induces hyperlipidemia and calcification of aortic lesions [179,180].

### 4.4. Limitation of In Vivo Models

The optimal choice of the animal model depends on the target to be assessed. A general limitation is that mice and rats are less prone to calcification than humans. In addition, a timespan of decades in humans where the disease progression occurs has to be limited for a study period of several weeks in animal models. Therefore, hard stimuli for induction might hamper the comparability to the human situation. However, animal models allow the study of signaling pathways under controllable conditions.

## 5. Conclusions

The variety of influencing factors and different components in the development of VC reflect, at least in part, the variety of research models and vice versa. Therefore, studying VC entails the challenge of utilizing a manageable experimental setting depicting the complexity of its pathophysiologic interrelations. In vitro models employ a variety of vascular cells and inducers of calcification for studying pathways and screening inhibitors and inducers; however, they provide a non-physiological environment. Ex vivo settings using vessel tissue meet this drawback at least partly and might bridge the gap to in vivo models. Animal models with rodents involve the induction of VC by establishing uremic conditions, genetically modified calcium–phosphate and lipid metabolism as well as impairment of calcification inhibitors. While offering a natural environment, immense interventions are needed to achieve the VC condition. The current data show that VSMC appear to be a central cell type within the mineralization process in the media of the vessel wall. Further research is required to understand the detailed mechanisms, contributors and inhibitors in the process of VC and to establish working treatment options to reduce and/or inhibit calcification and stiffening of the vessel wall leading to increased mortality. An ongoing effort should be taken to improve the experimental models studying VC by improving the identification and diagnostic tools for quantification of VC to achieve maximized comparability of the results. This might not only help to reduce animal numbers for primary cell isolation and in vivo settings in case of the 3R (Replacement, Reduction and Refinement) thought of Russel and Burch [181], but also identify the most promising therapeutic strategies to reduce the cardiovascular morbidity and mortality of the patients.

## Figures and Tables

**Table 1:** Selected cell types for researching vascular calcification in vitro.

Origin	Cell Type	Source
Tunica Externa	Myofibroblasts	[20]
Tunica Media	Primary VSMC	[21,22,23,24,25]
	MOVAS	[26,27,28]
	A7r5	[29,30]
	A10	[31,32]
Tunica Intima	Pericytes	[33]
	Endothelial Cells	[34]
Circulating	Mesenchymal origin	[35,36]
	Hematopoietic origin	[37,38,39]
Heart	Valvular Interstitial Cells	[40]

**Table 2:** Selected supplements and representative dosage for in vitro induction of calcification.

Supplement	Common Concentration	Source
Serum/FBS	0%–20%	[21,24,25,33]
Glucose	5–25 mM	[21,60]
Inorganic Phosphate: Sodium hydrogen phosphate	1.6 mM	[32]
Organic Phosphate: β-glycerophosphate	1.25–10 mM	[21,27,52]
Calcium	2.5 mM CaCl	[43]
Ascorbic Acid	10 µg/mL–50 µg/mL	[24,25,27,40]
Sodium pyruvate	10 mM	[21]
Insulin	10^−7^ M	[21]

**Table 3:** In vivo research models inducing vascular calcification in mice and rats.

Model Type	Predominant Localization of Calcification	
	Intimal	Medial
**Naturally Occurring**		DBA2 mice CY^+^ rat with autosomal dominant polycystic kidney disease Lewis polycystic kidney disease rat
**Operation**		Kidney reduction (electrocautery, nephrectomy)
**Feeding/Substance Application**	Cholesterol Rich Chow PCSK9-AAV	Adenine Vitamin D Phosphate Streptozotocin
**Genetic Modification**	Lipoprotein System *ApoE^−/−^* *Lldlr^−/−^* *ApoE3 Leiden* *PCSK9-AAV*	Phosphate Metabolism *Klotho^−/−^* *FGF23^−/−^* *Galnt^−/−^* *Tcal/Tcal* Pyrophosphate Metabolism *Abcc^−/−^* *Enpp1^−/−^* *Lmna^−/−^* Osteogenic Signaling *Fetuin A^−/−^* *Opg^−/−^* *Mgp^−/−^* *Opn^−/−^* *Madh6^−/−^*

Abbreviations: AAV: adeno-associated virus vector; Abcc6: ATP binding cassette subfamily C member 6; ApoE: apolipoprotein E; Enpp: ectonucleotide pyrophosphatase phosphodiesterase; FGF23: fibroblast growth factor 23; Galnt3: GalNAc transferase 3; MGP: matrix gla protein, OPN: osteopontin; OPG: osteoprotegerin; PCSK9: proprotein Convertase subtilisin/kexin type 9.

## Abbreviations

AA	Ascorbic Acid
ahsg	α_2_-Heremans-Schmidt Glycoprotein
AAV	Adeno-Associated Virus Vector
ALP	Alkaline Phosphatase
ApoE	Apolipoprotein E
Abcc6	ATP Binding Cassette Subfamily C Member 6
BUN	Blood Urea Nitrogen
BMP	Bone Morphogenic Protein
CKD	Chronic Kidney Disease
EC	Endothelial Cell
EndMT	Endothelial-to-Mesenchymal Transition
Enpp	Ectonucleotide Pyrophosphatase Phosphodiesterase
ECM	Extracellular Matrix
FBS	Fetal Bovine Serum
FGF23	Fibroblast Growth Factor 23
Galnt3	GalNAc trans-ferase 3
HDL	High Density Lipoprotein
hFTC	Hyperphosphatemic Tumoral Familial Calcinosis
Ppi	Inorganic Pyrophosphate
LDL	Low Density Lipoprotein
LDLR	Low Density Lipoprotein Receptor
LMNA	Gene encoding the Lamin A/C protein
MGP	Matrix Gla Protein
MMP	Matrix Metalloproteinases
Opn	Osteopontin
Opg	oPsteoprotegerin
PTH	Parathyroid Hormone
PCSK9	Proprotein Convertase Subtilisin/Kexin Type 9
ROS	Reactive Oxygen Species
RANK	Receptor Activated Nuclear Factor κB
RANKL	Receptor Activated Nuclear Factor κB Ligand
SPP1	Secreted Phosphoprotein 1
NPT2a	Sodium-Dependent Phosphate Co-Transporters Type IIa
NPT2c	Sodium-Dependent Phosphate Co-Transporters Type IIc
SD	Streptozotocin-induced Diabetes
TNFRSF11B	Tumor Necrosis Factor Receptor Superfamily Member 11B
VC	Vascular Calcification
VSMC	Vascular Smooth Muscle Cell
VLDL	Very Low Density Lipoprotein
